# A Critical Discourse Analysis of Indigenization in Saskatchewan's Undergraduate Nursing Programs

**DOI:** 10.1111/nin.70087

**Published:** 2026-02-06

**Authors:** Delasi Essien

**Affiliations:** ^1^ University of Regina Regina Saskatchewan Canada

**Keywords:** colonialism, critical discourse analysis, Indigenization, nursing education, racism, strategic plans

## Abstract

The nursing academy in Canada, motivated by the release of Canada's Truth and Reconciliation Commission's Calls to Action in 2015, has declared support for and commitment to Indigenization. This study, framed by the historical context of colonialism in Canadian healthcare and nursing education, aimed to understand the current state of Indigenization within undergraduate nursing programs in Saskatchewan. I explored three areas of inquiry: how strategic plans define Indigenization, staff experiences and practices in implementing it, and how these discourses and practices perpetuate or transform existing power structures. With Spivak's theory of the deconstruction of marginality as the overarching theoretical framework, I examined the strategic plans of three major post‐secondary institutions and their respective undergraduate nursing programs using Fairclough's dialectical‐relational approach to critical discourse analysis. I also interviewed a total of seven nursing staff members from the three programs to gain an understanding of their practices of Indigenization. The study identified four key constructs of Indigenization within the discourse of the province of Saskatchewan's nursing education strategic plans: Indigenous inclusion, relationship, reconciliation, and decolonization. A critical finding is that presenting these four constructs as interchangeable diminishes their unique, albeit interconnected roles in achieving Indigenization goals. The research further shows how nursing programs are centering marginalized Indigenous Knowledges and systems but also reveals that racism remains a barrier to Indigenization. The study emphasizes the importance of explicitly defining Indigenization and its goals to deconstruct power dynamics and practices within nursing programs, and to continuously examine racism as a persistent issue in nursing education. The study encourages the nursing academy to adopt a decolonial lens in its everyday Indigenization efforts, which will bring about transformation of the academy.

## 1 Introduction

The Indian Act of 1876, an active statute in Canada, served the colonial agenda of assimilation and control of Indigenous Peoples and the erosion of their land rights (Allan and Smylie 2015; Government of British Columbia 2020; Royal Commission on Aboriginal Peoples 1996). As a race‐based piece of legislation, the Indian Act, provided the Government of Canada with the legal and administrative framework for the establishment and operation of the residential school system in Canada, which was a systematic effort to assimilate Indigenous children into Euro‐Canadian society.

Since the closure of the last federally run residential school in Saskatchewan in 1996, residential school Survivors have spoken openly about their trauma and have pushed for acknowledgment and reparation (Hanson 2016; Stanton 2011). The launch of a class action lawsuit in 2005 by the Assembly of First Nations, with Grand Chief Phil Fontaine against the federal government, resulted in the Indian Residential Schools Settlement Agreement (IRSSA), which included the mandate for a Truth and Reconciliation Commission.

The Truth and Reconciliation Commission of Canada (TRC), whose objectives were to explore the experiences of Survivors of the residential school system in Canada and to lay the foundation for reconciliation, released its final report in 2015 (Truth and Reconciliation Commission of Canada 2015b). The report comprised 94 Calls to Action that called upon all sectors of society and government to acknowledge that colonial policies that have impacted Indigenous lives and health and to redress the historical legacy of cultural assimilation against Indigenous Peoples (Truth and Reconciliation Commission of Canada 2015b, 2015c).

The nursing academy in Canada, motivated particularly by the release of the TRC's Calls to Action, has expressed strong support for Indigenization with the aim of transforming relations between the academy and Indigenous Peoples (Canadian Association of Schools of Nursing 2023). To ensure that the obligations and commitments towards Indigenization are being met, it is imperative to understand the current state of Indigenization in nursing education, a decade after the release of the Calls to Action. With a focus on nursing education programs in the prairie province of Saskatchewan, Canada, I conducted this study to explore how Indigenization is represented in the language, practices, and experiences of nursing education and how the discourse of Indigenization sustains, reproduces, or transforms structures of dominance and power within nursing education programs (Essien 2025).

### 1.1 Social Positioning

My social identity as an Afro‐Canadian immigrant from a former British Crown colony affords me a uniquely empathetic perspective on the impact and legacies of colonization on Indigenous Peoples. However, I do not presume a shared experience of colonization and its subjectivities with the Indigenous Peoples of Canada and reject at the outset the supposition that Black and Indigenous experiences with racism and settler colonialism are interchangeable phenomena. I approached this research with humility as an outsider recognizing that I cannot truly “know” Indigenous cultures and sensibilities in the sense that such knowledge implies ownership. To this end, I reflected on what it means to be a settler of color—not just an immigrant in Canada but a settler on Indigenous lands who enjoys the rights and benefits of these lands. I examined and questioned the attitudes, thought processes, values, assumptions, and biases I brought to the study through reflexive practice as I critiqued the nursing academy—the very institution to which I belong.

## 2 Methodology and Methods

### 2.1 Fairclough's Dialectical‐Relational Approach to Critical Discourse Analysis

Fairclough (2016) posits that the methodology of the dialectical‐relational approach (CDA) is a theoretical process and has formulated an approach for using this methodology. Fairclough's (2016, 91) dialectical‐relational approach to CDA, described hereafter as Fairclough's CDA, is outlined in the following four stages:

- Stage 1: Focus upon a social wrong in its semiotic aspects.
- Stage 2: Identify obstacles to addressing the social wrong.
- Stage 3: Consider whether the social order “needs” the social wrong.
- Stage 4: Identify possible ways past the obstacle.

Fairclough's CDA is geared towards understanding the nature and sources of social wrongs, the obstacles to addressing those wrongs, and ways of overcoming those obstacles (Fairclough 2016). Social wrongs can be understood as aspects of social systems, forms, or orders which are detrimental to human well‐being, which could, in principle, be mitigated or eliminated through significant changes to these systems, forms, or orders.

The social wrong that the study addressed is the ongoing impact of colonialism in nursing education. With Indigenization as the focus, I examined the way the social systems—the nursing education programs—are structured or organized to prevent addressing the identified social wrong. I drew primarily on Spivak's (2009) theory of the deconstruction of marginality, which allowed me to examine Indigenization as a process embedded within power relations in the academy. Spivak's theory is undergirded by other theoretical frameworks including Foucault's (1975) theory of knowledge and power. The study's findings are discussed using the four stages of Fairclough's CDA to analyze the state of Indigenization in the three undergraduate nursing programs in Saskatchewan.

### 2.2 Overview of the Study

I analyzed the strategic plans from the University of Saskatchewan, the University of Regina and Saskatchewan Polytechnic and their respective undergraduate nursing programs in the prairie province of Saskatchewan, Canada. I also examined relevant texts that were referenced in or emanated from these strategic plans (see Table 1). Participants were recruited from the three undergraduate nursing programs via print and electronic fliers distributed between January and May 2024. The participant inclusion criteria were the following: (a) be an administrator, faculty member, instructor, or staff of one of the undergraduate nursing programs in Saskatchewan and (b) been involved in or engaged with implementing Indigenization strategies in the program(s).

**Table 1:** Documents analyzed from the three institutions.

Institution	Source of document	Name of document (publication/retrieval date)
University of Saskatchewan (USask)	Parent institution	University Plan 2025 (published in 2018) 2025 Aspirations (webpage retrieved on January 17, 2022)
	College of Nursing	Plan 2025 College of Nursing Strategic Plan (published in 2018) College of Nursing—Indigenous Initiatives (webpage retrieved on February 23, 2022) 2025 Strategic Plan presentation (published in September 2018)
University of Regina (U of R)	Parent institution	Strategic Plan 2020–2025 (published in 2020) Statement of Commitment in Response to the Truth and Reconciliation Commission (published in December 2018) A Guide to Implementing the Truth and Reconciliation Commission of Canada's Calls to Action at the University of Regina (published in December 2018)
	Faculty of Nursing	Faculty of Nursing 2020–2025 Strategic Plan (published in June 2021) Faculty of Nursing website landing page (published in 2021)
Saskatchewan Polytechnic (Sask Polytech)	Parent institution	Strategic Plan 2020–2025 (published in 2020) Academic Model (published in 2016) Indigenization Declaration (published in 2015)
	School of Health Sciences and School of Nursing	Strategic Initiatives—School of Health Sciences and School of Nursing 2020–25 (published in 2020)

#### 2.2.1 Data Collection

The strategic plans and documents examined were publicly available and accessed through the institutions' websites and program‐specific webpages. Seven participants also provided informed consent to be interviewed about their experiences with Indigenization. Six semi‐structured interviews were conducted in May 2024, comprising five individual sessions and one paired session. An interview guide provided consistency across the sessions, which lasted approximately 60–90 min each. The interviews were conducted via Zoom, audio‐ and video‐recorded, transcribed verbatim, and subsequently coded and analyzed using NVivo 15 software.

All the participants were female and were either program support staff or faculty with experience in implementing Indigenization strategies. Three of the participants identified as Indigenous, three of them identified as having European roots, and one identified as Black. Overall, there were at least two participants from each of the three undergraduate nursing programs.

#### 2.2.2 Ethical Considerations

The study received harmonized Research Ethics Board approval through the University of Regina (REB # 2021‐035), with reciprocal approvals through the University of Saskatchewan and Saskatchewan Polytechnic. Participants were informed that participation was voluntary, with the right to withdraw at any time without consequence and the ability to skip any questions they found uncomfortable. To maintain anonymity and confidentiality, transcripts were de‐identified, and participant names were replaced with pseudonyms during analysis. All data were stored electronically in password‐protected files. Participants were also given the opportunity to review their interview transcripts and provide feedback through a secure, password‐protected electronic exchange.

I engaged in critical reflexivity, in keeping with recommendations by Berger (2015), by examining my positionality as a Black nursing educator and administrator; examining how my epistemological assumptions, social identity—ethnicity, social class, gender, and race—and experiences influenced the research process and interpretation. To capture this, I documented my internal dialogues to reflect on my situatedness, personal investments, and potential biases.

#### 2.2.3 Data Analysis

I utilized a content analysis process adapted from Lune and Berg's (2017) stage model of qualitative content analysis to examine the strategic plans and the interview transcripts. Then, I applied Fairclough's (2016) dialectical‐relational approach to critical discourse analysis (CDA) to the findings. CDA methodology as a whole has been criticized for its subjectivity (Essien 2025). However, juxtaposing Fairclough's CDA with Lune and Berg's (2017) content analysis method—an analytical tool that emphasizes objectivity—made the CDA approach a more trustworthy and rigorous one.

#### 2.2.4 Trustworthiness and Rigor

The research process was assessed for credibility, dependability, conformability, auditability, and transferability (Elo et al. 2014; Lincoln and Guba 1985) to ensure rigor and trustworthiness. Credibility and dependability were established through the use of continuous reflexivity to ensure that interpretations were grounded in the data. Code descriptions were used to maintain consistency in coding process, interpretations were corroborated with data extracts, and member checks were used to verify with the participants that the information captured in each interview was accurate. Representative quotes from the transcripts are presented to support auditability. An audit trail in the form of process journals and a code book was used throughout the study to support auditability, conformability, and transferability of the results. A theoretical sampling method was used, and the process for achieving theoretical saturation was detailed to increase the trustworthiness of the study.

### 2.3 Literature Review

Many Canadian post‐secondary institutions (PSIs) have adopted Indigenization as a strategic priority (Gaudry and Lorenz 2018). Consequently, Indigenization has been brought into institutional focus through the use of strategic plans. However, despite the plethora of definitions of Indigenization, there is no consensus on a singular definition by these PSIs.

#### 2.3.1 Definitions of Indigenization

Indigenization describes the process of challenging the dominance of Western thought and braiding it with Indigenous Knowledge systems to allow for the embodiment, production, and practice of Indigenous Knowledges in typically colonial or Western spaces (Essien 2025; Grafton and Melançon 2020; Harder et al. 2018; Smith et al. 2019). It signifies the valuing and acceptance of the legitimacy of Indigenous Knowledges and epistemologies and the influence of Indigenous scholars within the academy and re‐centers marginalized Indigenous Knowledges at the core of instructional practice (Docherty et al. 2023; Efimoff 2022; Essien 2025; FitzMaurice 2011; Grafton and Melançon 2020; Harder et al. 2018; Pete et al. 2013).

Scholars have described the work of Indigenization in higher education as being focused on sincere reciprocal relationship building with Indigenous Peoples and communities, strengthening cultural identity of Indigenous learners, ensuring Indigenous rights to self‐determination, confronting racism and exposing whiteness, and seeking to overcome oppression by transforming the academy (Biin et al. 2021; Grafton and Melançon 2020; Pete et al. 2013). Indigenization at Canadian PSIs has taken the form of Treaty land acknowledgments, Indigenous artworks that tell stories or represent visual histories, the inclusion of Indigenous Knowledge into curricula, and the hiring of Indigenous faculty. Other examples include the recruitment and retention of Indigenous students; the introduction of access or bridging programs, tutorials, student support services that are intended to increase Indigenous graduates; Indigenous‐specific scholarships; creating new Indigenous spaces; hosting Indigenous events; and developing broad‐based institutional policies about Indigenizing campuses (Brunette Debassige et al. 2022; Efimoff 2022; FitzMaurice 2011; Thurston and Mashford‐Pringle 2015).

In nursing education programs, the processes and actions of Indigenization—which mirror those of many PSIs—have been criticized by some nursing scholars. For example, Bourque Bearskin et al. (2022) caution that Indigenization in nursing programs can become misguided performative benevolence and paradoxically contribute to recolonizing, tokenizing, and erasing Indigenous Knowledges and Peoples. Other critics have indicated that Indigenization initiatives in nursing education are inherently colonial because the nursing profession itself is based on a colonial system of values (McGibbon et al. 2014).

These criticisms highlight that Indigenization must go beyond inclusion policies in nursing programs to effectively challenge colonial systems that have marginalized Indigenous Peoples and Knowledge. This is perhaps where the work of Indigenization intersects with decolonization. The transformation that comes through Indigenization has been described as one that decolonizes oneself and the collective work of academics (Docherty et al. 2023; Gaudry and Lorenz 2018; Grafton and Melançon 2020; Pete et al. 2013). As a decolonizing process, Indigenization is a shared responsibility for Indigenous and non‐Indigenous peoples and necessitates relational accountability to the Indigenous People who are part of the collaborative process (Fraser 2022; Grafton and Melançon 2020).

## 3 Findings and Discussion

To employ Fairclough's methodology, I restated these four stages as questions to semiotically analyze and evaluate the discourse of Indigenization in the nursing education programs in Saskatchewan. The questions corresponding to each stage of the methodology were the following:

- What is the social wrong the study focuses on?
- What obstacles to addressing the social wrong were identified in the discourse?
- How have the nursing education programs resisted the social wrong?
- What are the possibilities of overcoming the social wrong in the programs?

### 3.1 The Social Wrong

As previously stated, the social wrong that the research focuses on is the ongoing impact of colonization in nursing education. The impacts of the many forms of colonialism in Canada (see Essien 2025) are far‐reaching, persisting across generations and shaping the dynamics of the relationship between Indigenous and non‐Indigenous people in Canada. And nursing, as a professional discipline, is not removed from this colonial history. In fact, nursing has been implicated in perpetuating Indigenous‐specific racism and advancing the ongoing disparities in health for Indigenous Peoples (Essien 2025; Hantke 2022; Puzan 2003). Therefore, to understand the discourse of Indigenization, I situated the discourse within the colonial healthcare context in Canada.

Colonization has been identified as a foundational determinant of Indigenous health (Allan and Smylie 2015; McCallum 2017) and is interrelated with racism. Canada's practice of race‐based legislation has had a lasting effect on Indigenous identity, health, and well‐being. This is evidenced by lower life expectancy and higher incidences of diabetes and co‐morbidities, infant mortality, depression, and suicide in Indigenous Peoples in comparison to non‐Indigenous peoples in Canada (Allan and Smylie 2015; Browne et al. 2016; Swidrovich 2022; Truth and Reconciliation Commission of Canada 2015a). Contemporary scholarship reveals that Indigenous Peoples became the target of genocidal practices backed by policies such as, but not limited to, the Indian Act, the Indian hospitals, the Indian Residential Schools, and the *Sixties Scoop*.^1^ These policies contributed to anti‐Indigenous racism developing roots in healthcare.

Colonial ideals embedded within systems like child welfare and healthcare were (re)produced by professionals, who viewed their everyday practices to be in the best interest of Indigenous Peoples. Some saw themselves as providers of what they imagined Indigenous Peoples were lacking, that is, intelligence, work ethic, or parenting skills (Allan and Smylie 2015; Gebhard et al. 2022; Truth and Reconciliation Commission of Canada 2015d). By assuming the role of innocent altruists who simply wanted to help, healthcare professionals, including nurses, became instrumental in colonial projects. These colonial projects included intentional infection with smallpox, mass medical evacuations of Indigenous People to tuberculosis sanatoria, forced female sterilization programs, nutritional experiments, abusive surgical procedures, and enforcing assimilationist policies in residential schools and Indian hospitals (Daschuk 2019; Government of British Columbia 2020; Hantke 2022).

Although many of the more overt forms of colonialism have been abolished, colonial mimicry has been institutionalized in systems, such as nursing education. Colonizing beliefs and assumptions, including intersections of white privilege, discrimination, and epistemic racism, form the foundation for colonialism in nursing (McGibbon et al. 2014). Dominant discursive practices in nursing have emerged and continue to operate within a frame of these colonizing ideologies. As a result, colonial contexts and anti‐Indigenous racism are reproduced, encultured, and coached into nursing theory and praxis. For example, some regulations within the Indian Act did not recognize or give credence to Indigenous Knowledges and practices of healing and traditional medicines, forcing the acceptance of Western medicine and health practices for the sake of accessing healthcare (Meijer Drees 2013). This ideology reverberates in present‐day discourses of evidence‐based practice in nursing—where commitment to the hegemonic discourse of science has often been upheld to the exclusion of Indigenous healing practices as legitimate (Puzan 2003; Swidrovich 2022)—as a form of epistemic violence.^2^

The ongoing impacts of colonization in nursing education are not a problem just for Indigenous Peoples. The sordid history of nursing education's complicity in perpetuating colonial violence and savagery is a history that every nurse who passes through the institution of nursing comes to share. TRC's Call to Action #24 is explicitly addressed to nursing (and medical) schools in Canada:

We call upon medical and nursing schools in Canada to require all students to take a course dealing with Aboriginal health issues, including the history and legacy of residential schools, the United Nations Declaration on the Rights of Indigenous Peoples, Treaties and Aboriginal rights, and Indigenous teachings and practices. This will require skills‐based training in intercultural competency, conflict resolution, human rights, and anti‐racism.

(Truth and Reconciliation Commission of Canada 2015a, 3)

The fact that nursing education programs in Canada are specifically called out and called upon is telling and speaks to the necessity to redress the legacy and mitigate the impacts of colonialism in these programs. It is imperative for the nursing academy to be a place of physical, psychological, and cultural safety for both Indigenous Peoples who are dealing with direct or intergenerational traumas and for non‐Indigenous people who are learning to (re)build relationships with Indigenous Peoples. And this is the responsibility of both Indigenous and non‐Indigenous peoples alike. In the words of Sochan (2011), “nurses must not only recognize that it is within their power to challenge disciplinary colonization, they have an obligation to actively engage in decolonizing actions in order to begin reversing these effects” (p. 185).

Although not explicitly mentioned in the Calls to Action, Indigenization is one of the ways by which the nursing academy has responded to the scrutiny of the TRC. Commitments to Indigenization are laid out explicitly in the strategic plans of the nursing programs in Saskatchewan. And while strategic plans on their own do not have agency, they inform and encourage actors to coordinate programmatic activities by making explicit what is prioritized, what is included or excluded, and what is visible or invisible in the experiences and practices within the institutions. This is why strategic plans and interviews of staff experiences in implementing Indigenization were the focal points of entry into examining how the impacts of colonization are being redressed within the programs as well as what the obstacles are to addressing the social wrong.

### 3.2 Obstacles to Addressing the Social Wrong

Through the analysis, Indigenization was explicated as consisting of four interrelated and interdependent constructs/themes. These four themes—Indigenous inclusion, relationships, reconciliation, and decolonization—are deeply embedded within and constitutive of each other and shape the understanding of Indigenization in the nursing programs in Saskatchewan.

Indigenous inclusion manifests in the strategic plans as creating space, incorporating Indigenous ways of knowing, and providing support for Indigenous students, faculty, and staff. This finding is consistent with Gaudry and Lorenz's (2018) characterization of Indigenization as Indigenous inclusion. They assert that Indigenous inclusion is a policy‐based agenda that aims to increase the number of Indigenous students, faculty, and staff in an already established Western academic structure by supporting the adaptation of Indigenous People to the current, often alienating culture of the Canadian academy (Gaudry and Lorenz 2018). However, the study's findings showed that the three institutions are challenging this narrow definition of Indigenous inclusion.

The activities of Indigenous inclusion listed by the three institutions in their strategic plans—creating space, incorporating Indigenous ways of knowing, and providing support for Indigenous students, faculty, and staff—suggest that in addition to attempting to increase the numbers of Indigenous students, faculty, and staff, the three institutions are also opening and encouraging Indigenous worldviews, knowledges, and experiences to occupy space within the academy. For nursing academics, staff, and administrators who are open to Indigenous perspectives and are challenging normative ones, Indigenous inclusion, as characterized by the three institutions, can be a catalyst for critical reflection and shifting perspectives; thus, exemplifying an orientation towards transformation.

Second, Indigenization is described in the strategic plans as a commitment to be in good relationship with Indigenous people as characterized by treaty land acknowledgment, collaborating and engaging with Indigenous people, mutuality, and reciprocity. The third theme, Indigenization as reconciliation, is an interdiscursive interplay of how the responsibility to reconciliation and the response to the TRC's Calls to Action are articulated together. Although non‐Indigenous people are encouraged to follow the lead of Indigenous People, the three institutions lean towards reconciliation as primarily a responsibility of non‐Indigenous people. The fourth theme, decolonization, is semiotically linked to both Indigenization and reconciliation in the strategic plans. The adjective used to describe decolonization is *transformative*. This is apt given that decolonization challenges the status quo and demands transformation. Decolonization requires reflexivity, autocriticism, and critical scrutiny from administrators, staff, and faculty of the nursing education programs into actions, frameworks, policies, and programming to intentionally remove any colonial elements and create space to include previously excluded Indigenous elements.

Although not exclusive of each other, these four constructs of Indigenization represent different processes and require unique approaches. However, two significant observations were made in the analysis of the strategic plans: (1) the constructs of Indigenization were conflated and presented as interchangeable processes, and (2) reconciliation and decolonization were vaguely described all three undergraduate programs even though they featured prominently in the strategic plans.

To illustrate the first point, activities described as Indigenization by the University of Saskatchewan's and Saskatchewan Polytech's nursing programs are categorized under reconciliation in the University of Regina nursing program's strategic plan. Notably, this conflation was also observed in the extant literature review and in the participant interviews. One participant, on separate occasions during the interview, defined Indigenization, reconciliation, and decolonization as reclaiming Indigenous identity. She stated:

[Decolonization] means reclaiming our identity … our ways of knowing and our culture… reclaiming it or continuing on with it. So, in that regard then if we're reclaiming our identity, or celebrating and moving on with it, we're Indigenizing it. …And so, reconciliation is living your Indigenous identity in a positive way.

(Participant 1)

Additionally, the documents examined offered no clear definitions of reconciliation or decolonization. To buttress this point, there was only one definition of decolonization found in the documents analyzed in the study. In their strategic plan, the University of Saskatchewan (2018) describes decolonization both as practices that contest divisive and demeaning actions, frameworks, policies, and programming, as well as a process which is amplified by Indigenization and leads to reconciliation. This vague description of decolonization further compounds the conflation.

When the terms Indigenization, relationship, reconciliation, and decolonization are used interchangeably by nursing education programs or are intentionally left vague in their strategic plans, it creates ambiguity in the meaning of the individual constructs. Consequently, this ambiguity detracts from how each of the constructs individually and collectively supports the goals of Indigenization and becomes an obstacle to addressing the social wrong. This problem is further explained using Foucault's (1975) power/knowledge couplet and Spivak's (2009) theory of the deconstruction of marginality.

Using the concept of power/knowledge as a couplet, Foucault (1975) theorized that the development of new forms of knowledge—those imbued with power and expressed through discourse—creates broader and deeper understandings of the social and physical world, thereby simultaneously generating new sites for the application of power. However, for Foucault, knowledge is always already deeply invested with power and is never separate from power (Appelrouth and Edles 2016). Thus, power is most potent when it is successfully translated into systems of knowledge in such a way that it becomes taken for granted (Appelrouth and Edles 2016).

Spivak (2009) borrows from Foucault's power/knowledge couplet to underpin the theory of the deconstruction of marginality. According to Spivak (2009), there is an academic center within each institution which constitutes power/knowledge. In this academic center, boundaries are created around what is prioritized, is included, or excluded, and made visible or invisible. Indigenization, which has not been traditionally part of institutional language, has been brought into an academic center that “relies on traditional and inherited power structures and knowledge formation to function” (Jackson and Mazzei 2023, 43) by way of institutional strategic plans. Therefore, how Indigenization is positioned in the center is a function of how knowledge articulates with power within the halls and walls of the academy: who has the power to be a knower and whether their knowledge is commensurate with, Moreton‐Robinson (2015) posits, “the West's ‘rational’ belief system” (p. 13).

In other words, Indigenization—positioned as a dominant discourse in the nursing education programs—has become a consequence of power and a mechanism to exert power. And the four constructs of Indigenization, identified through the strategic plans, have become technologies of the power/knowledge center by shaping and coordinating the activities of Indigenization within the institutions. Consequently, any ambiguity associated with these constructs becomes a taken‐for‐granted reality that translates into day‐to‐day academic work. This was evident in the study when none of the participants could clearly articulate how they were demonstrating reconciliation and decolonization as part of their overall goals of Indigenization. For example, one participant shared:

When I do think of reconciliation and decolonization, it's a response to something negative that happened. And they don't want to always be reacting and responding to something negative. Although it's a really—it's a real part of our history. I don't want to say that those things never happened. They did happen. And it's important that we realize it … Indigenization is the most positive of all these words that are floating around. It's not a response to something negative that happened. But it's a celebration of the strengths and the mental strength, the physical strength, the spiritual strength, and the emotional strength of your culture. … That's what Indigenization means to me.

(Participant 1)

The findings showed that having no concrete definitions of reconciliation and decolonization in the strategic plans negatively impacted how those concepts translated into practice.

### 3.3 Resisting the Social Wrong

In Stage 3 of Fairclough's CDA, the focus of the analysis starts to shift to the positive critique of addressing and mitigating the social wrongs by scrutinizing the social order itself—the nursing education programs in Saskatchewan. I recognize that Indigenizing efforts can serve as a facade while maintaining the status quo of Eurocentric hegemony. However, the fact that Indigenization is addressed in some form in the strategic plans may be an indication of willingness by the institutions to disrupt the status quo. As one participant stated, “what is working well is people are talking about [Indigenization]. Because when I first moved to Saskatchewan, no one was talking about it” (Participant 4).

During the interviews, participants reflected on what would be required for the necessary change to happen, which included building relationships with Indigenous communities and peoples. They were also able to identify actions that would hinder and detract from Indigenization efforts such as performative actions that had no material consequences to advancing Indigenization, for example, rote recitals of land acknowledgment. Hopeful about the ability of the nursing programs to resist the social wrong and bring about transformation, one participant stated:

If it's done right … I think [Indigenization] should become the norm. So, we're not even realizing that we're Indigenizing something or decolonizing … Like I used the example of ribbon skirts. It wasn't that long ago if you wore that you wouldn't have been allowed to go out in public. You know, we wouldn't have seen people's regalia. Now people can do their ceremonies. They can do their dances. They can wear their stuff with pride. … 30 years ago, that wouldn't have happened and now we don't even think twice if we see somebody in the halls with a ribbon skirt or earrings, right? So that's what I'm saying. It's kind of becoming the normal.

(Participant 3)

This hope for Indigenization in the nursing academy in Saskatchewan is consistent with the goals of Indigenization in the literature—to (re)center Indigenous resurgence, sovereignty, worldviews, voices, and experiences (Alfred 2018; Essien 2025).

However, it is important to note that not all participants' views projected a hopeful and positive image of Indigenization. It became apparent through the interviews that racism is an ongoing issue that poses a challenge to Indigenization efforts within the nursing programs. One Indigenous participant described multiple instances of racism Indigenous faculty face including not being allowed to attend Indigenous events because their non‐Indigenous colleagues complained about the extra time Indigenous faculty need to participate in cultural activities, and being told by their non‐Indigenous colleagues that they only got their job because of their Indigenous heritage (Participant 3). Another Indigenous participant opined that being burdened with the responsibility of educating their non‐Indigenous colleagues about “everything Indigenous” was problematic (Participant 2). Burdening a single individual with educating other program staff on Indigenous ways of knowing and being requires that the individual have an impossible breadth of knowledge, as well as time to do so. Raffoul et al. (2022) argue that placing such responsibility on Indigenous faculty and staff runs counter to the emphasis of relationality in many Indigenous cultures and adds to racist attitudes of “tasking the colonized with educating the colonizer” (p. 167).

According to Gebhard (2022), White faculty are more likely to deny that the problem of racism exists, but they also tend to contradict this claim with narratives that reveal how they and their colleagues harbor racist assumptions that in turn, shape their practices. This was evident in the study when a non‐Indigenous participant—who insisted they were “not a racist”—had this to say about the Indigenous nursing students in their program:

So, we don't do well across the country because our NCLEX^3^ rates aren't high for passing because we're … we've got so many Indigenous students … I mean there's some passing through nursing with 52%–53%. They never pass their NCLEX. And who do you want to be your nurse in the hospital in ICU? Do you want somebody that's passing with 52% or do you want somebody that's passing with 93%? I'll tell you who I want for my nurse … It just frightens me to death.

(Participant 5)

The same participant also made these comments about the Indian Residential School system:

I've had a lot of Indigenous People say, well, “the residential schools are the best thing that ever happened to me because I was in such a dysfunctional abusive family that it took me out of being sexually abused and beaten with addictions and everything else in the family” … I think a lot of Indigenous People think that the atrocities only happened to them in the world… We've always had somebody at the bottom of the totem pole. And if it is not native people, it is women… You'd think that our society has matured a bit, but we're not. Look at Ukraine. Look at Israel and Palestine … like, my God! They're still killing each other. Residential school is a lot better than killing each other in wars, you know.

(Participant 5)

These comments and experiences, shared by the participants, expose some of the ideologies that contribute to the broader discursive practices of Indigenization in the nursing programs in Saskatchewan. The negative constructions of Indigeneity contribute to social conditions where the lives of Indigenous People are less valued (Nunn 2018) and provide the “ideological justification for the ongoing dispossession of lands, the accompanying attack on sovereignty, and the maintenance of cultural imperialism” (Cannon and Sunseri 2018, xvi). In nursing programs, these sets of beliefs and thought patterns can be transmitted or coached into nursing students through a process described by Lavallee and Harding (2022) as Indigenous‐specific racism coaching. Pervasive discourses such as those that (re)produce Indigenous students as uninterested in academics, anti‐school, and/or having learning difficulties perpetuate racist colonial scripts and Indigenous‐specific racism.

“Not racist” neutrality—the denial that one is a racist—has been described as a “mask for being racist” by Kendi (2023, 19), who further opines that the opposite of “racist” is not “not racist” but rather “antiracist.” To be antiracist involves understanding how the present exists upon colonial and racist foundations; actively identifying, challenging, preventing, and eliminating racism from our values, structures, policies, programs, practices, and institutions; committing to educate oneself and others; and taking action to create conditions of greater inclusion, equality, and justice (Dordunoo et al. 2024; Government of British Columbia 2020; Hantke 2022; Kendi 2023).

### 3.4 Overcoming the Social Wrong

The final stage of Fairclough's CDA involves developing a semiotic point of entry into the ways that the obstacles to addressing the social wrong are formally or informally tested, challenged, and resisted within the social structure (Fairclough 2016). The document analysis showed that the nursing programs in Saskatchewan have each identified tangible steps towards actualizing the commitments that have been made to Indigenization within the programs. These steps were listed as actions, key performance indicators, or statistical reports and measures of Indigenization's progress (see Table 2).

**Table 2:** Key steps undertaken towards indigenization by the nursing programs.

Institution	Actions taken
University of Saskatchewan (College of Nursing 2022)	The College of Nursing has created a landing page on its website titled “Indigenous Initiatives,” highlighting progress made towards the ongoing journey in reconciliation. The webpage details ways the College has responded to the TRC's Calls to Action numbers 7, 22, 23, and 24. These include increasing Indigenous student representation and access to graduate‐level programs, incorporating Indigenous concepts such as the Medicine Wheel and other traditional ways of knowing into the curriculum, as well as recognizing postcolonial understanding, cultural competency, and inclusivity as core competencies that graduating students must obtain. The College of Nursing also boasts of eliminating the gap in participation in nursing education between Indigenous and non‐Indigenous students by establishing 16.6% equity seats for Indigenous students and having the highest number of enrolled Indigenous students in a nursing program across the country in the fall semester of 2020.
University of Regina (Faculty of Nursing 2021)	The Faculty of Nursing has delineated 16 actions to deliver on the commitment to truth and reconciliation within the faculty. They include strengthening relationships with Indigenous communities, creating principles and opportunities for engagement with Indigenous individuals and communities, and creating opportunities for all learners to engage with Indigenous ways of knowing and being. These 16 actions are grouped broadly into three overarching goals, namely, (a) improve supports for Indigenous students, faculty, and staff; (b) provide educational opportunities and experiences across Saskatchewan; and (c) incorporate Indigenous ways of knowing into teaching and research.
Saskatchewan Polytechnic (School of Health Sciences and School of Nursing 2020)	The Schools of Health Sciences and Nursing list the following as actions to fulfill the commitment to truth and reconciliation: (a) implement the Academic Model 1.5 project^a^ into programming; (b) construct an Indigenous Advisory Council in the Schools to promote and support Indigenization within all programs and to provide guidance to the Schools' leadership; (c) develop and deliver an Indigenous student recruitment and support strategy for the Schools; and (d) develop and deliver an Indigenous faculty and staff recruitment strategy for the Schools. The key performance indicator is stated as “increasing inclusion of Indigenous People in the learning and work environment” (School of Health Sciences and School of Nursing 2020, 5).

^a^ Saskatchewan Polytechnic's Academic Model is a framework that guides the design and delivery of programs in the institution. The framework is made up of five elements with 29 components. Project 1.5 addresses how the institution will meet the promises made towards Indigenization (Saskatchewan Polytechnic 2016).

It is noteworthy that these steps identified by the three institutions all mostly fall within the parameters of Indigenous inclusion. And while Gaudry and Lorenz's (2018) research has shown that Indigenous inclusion policies have had a beneficial impact on Indigenous People in the academy, they also argue that Indigenous inclusion allows for very little transformation of the academy itself. The study, however, revealed an interesting finding in this regard.

The study showed that while all three programs leaned more towards activities that are characteristic of Indigenous inclusion in their strategic plans, participants leaned more towards building relationships as the gateway to Indigenization. By prioritizing respectful and authentic relationship building with Indigenous communities and peoples, the participants reported on how they are orienting their approaches to Indigenization towards *decolonial Indigenization*. These included changing the lexicon of the program from *Aboriginal* to *Indigenous*, spearheading changes to how Indigenous students are honored during convocation, pioneering the creation of unique Indigenous elective courses for the nursing programs, and changing approaches to student interactions to make them more family‐ and community‐oriented. Six of the seven participants confirmed that they had made some headway in building relationships with Indigenous Peoples and/or communities and suggested this was the path towards Indigenization. This observation exposed how transformation of the academy may be happening on a relational level.

Furthermore, this finding is consistent with the conclusion that Gaudry and Lorenz (2018) draw at the end of their study:

We conclude that despite using a language of reconciliation, in practical terms the Canadian academy still largely focuses on policies of inclusion. In contrast, Indigenous faculty, staff, students and their allies are much more likely to envision a fundamental and decolonial shift.

(p. 226)

Consequently, the study reveals an important paradox: the overall discourse of Indigenization in the undergraduate nursing programs in Saskatchewan simultaneously sustains, reproduces, and transforms power structures. Transformation is essential to remedy the past, contextualize the future, and ensure the stability of future generations (Raffoul et al. 2022). For Indigenization to bring about significant transformation within the academy, it needs to be a shared vision. In the words of Gaudry and Lorenz, “there is a place for everyone to build this vital future” (p. 226). Working together to create a climate where transformation is possible is the responsibility of every member of the academic community.

## 4 Implications for Practice

At the conclusion of the study, there were three key takeaways: (1) How Indigenization is defined by the nursing programs is important for its implementation within the programs; (2) Opportunities exist within the prevailing discourse of Indigenization in the nursing programs to examine how Indigenization has been value‐coded, who and what are in the center and margins of the discourse, and how the center can rupture to give way to something new; and (3) Racism is still an ongoing issue in nursing programs despite Indigenization efforts and needs to be addressed.

### 4.1 Importance of Defining Indigenization

The analysis of the strategic plans shows that although Indigenization in nursing education programs represents four distinct constructs—Indigenous inclusion, relationships, reconciliation, and decolonization—these terms are used interchangeably to represent Indigenization. The analysis of the interview transcripts also shows similar conflation in what Indigenization represents. When these constructs are conflated and presented as interchangeable processes in the discourse of Indigenization, nursing education programs run the risk of de‐emphasizing each of these constructs as unique processes in actualizing Indigenization in the academy.

The study offers institutions a valuable heuristic to examine how Indigenization can be brought into the academic center in a meaningful way vis‐à‐vis strategic plans. I propose that in the next iterations of the strategic plans, Indigenization and its goals be clearly defined through a participatory process that incorporates the voices of Indigenous nursing faculty, staff, and students. Indigenizing the academy without Indigenous leadership can lead to new forms of cultural appropriation (Raffoul et al. 2022) and can exemplify epistemic violence. The conceptual framing of Indigenization, its practice, and its application should be expanded on to remove the ambiguity in defining what Indigenization is, with the help of the local Indigenous community on and off campus.

### 4.2 Opportunities for Change

This analysis of Indigenization, based on Spivak's (2009) theoretical premise, is not merely a critique of the meaning of Indigenization or a substitution for another truth of what Indigenization should be, nor is it simply a process of re‐inscription of Indigenization. Rather, it is a search for an irruption—a destabilization of seemingly fixed categories in ways that prevent a closure of meaning. When the members of the nursing academy actively ask questions that impugn the very foundations of entrenched processes and practices, it opens up the academic center to recode what is valued and destabilize what is fixed.

However, it should be noted that the center is not an empty signifier without actors. There are people within the academic center who are the gatekeepers of the academy's power/knowledge couplet—the power and knowledge structures that ensure that things are done the same way they have always been done whereby the status quo is maintained. These gatekeepers, Moreton‐Robinson (2015) states, are those who have the power to constitute what knowledge is valid. As these gate keepers—nursing administrators and their delegates—plan for next iteration of the strategic plans, I encourage them to ask the question “What needs to change in the way we have always done things to allow Indigenization to thrive in the institution and the program?” This question gets to the center of the power/knowledge couplet of the academy and accelerates the process of rebalancing the sources and repositories of power in the center. This question needs to be asked many times during the lifecycle of the strategic plans. It is also imperative that nursing educators be more than just allies. “It is time for them to be accomplices, drawing on their own power and privilege to challenge dominant systems” (Raffoul et al. 2022, 170).

The rupture of the center will involve dismantling governance systems. This is important because the hierarchy of relationships, as determined by the Western structure of governance, does not align with Indigenous worldviews on relationality (Snow et al. 2024). Given the axiological differences between the colonial academy and Indigenous communities, honoring and giving credence to Indigenous Knowledges and voices will be foundational for the success of bringing Indigenization into the academic center. Indigenous voices are amplified as Indigenous People and their allies participate in discursive practices that allow for both Indigenous and non‐Indigenous academics to challenge dominant taken‐for‐granted practices that have shaped power structures within the academy.

### 4.3 Addressing Racism in the Nursing Education Programs

Although not reported by every participant, instances of racism were identified by at least one participant in each of the three undergraduate nursing programs in Saskatchewan. Notably, there was only one participant who expressed negative views of Indigenous People. However, research has shown that people who hold views that might be considered racist or bigoted by others very seldom speak up for fear of appearing racist (McLean 2022). The comments and lived experiences of racism that surfaced during the interviews suggest that Indigenous‐specific racism exists in the undergraduate nursing programs, and sheds light on some of the barriers to implementing Indigenization within the programs.

The study is a reminder that colonialism—as racism—is still functional within this highly regarded and benevolent profession of nursing. This study reinforced that settler colonialism is not an event but rather a structure (Wolfe 2006); otherwise, Canada's colonial past would have stayed right there, in the past. According to Cannon and Sunseri (2018), racism cannot be reduced to individuals alone, and in racial terms, the enemy is not even the “white man” (p. 1). Rather, they suggest that racism is a way of thinking with an imperialist mindset that requires institutional analyses centered on racism's ideological basis and underpinnings to combat it. This study has exposed that there is more to be done by way of antiracism education and interventions in nursing education programs in Saskatchewan.

According to Lavallee and Harding (2022), too often Indigenous‐specific education in healthcare focuses on cultural differences instead of racism, which alone risks creating and reasserting stereotypes. In contrast, antiracism education produces a representation of Indigenous People as human beings who have been subjected to socially constructed inferiority and holds those that perpetuate colonialism accountable to learn and do better (Hantke 2022). Nurses must consciously build antiracism into their practices by unlearning biases that render Indigenous Peoples—and other racialized people—as inferior. Antiracism education needs to be accompanied by the historical and colonial contexts and allow for reflection on a person's positionality in relation to historical and ongoing colonial oppression. It is in these spaces of dialogue and critical reflection that one may recognize the deficiencies in one's viewpoint and perspectives (Mezirow 1978)—recognitions that can lead to the transformation of the nursing academy.

## 5 Conclusion

Informed by the literature and enlightened by the data analyses of the strategic plans and interviews that comprised this study, I suggest that Indigenization happens along a continuum of individual and collective learning journeys. I argue that on one end of this continuum, the academy supports and actively participates in oppressive practices; and on the farthest end, the academy is completely transformed as the Eurocentric center gives way and ruptures to allow for a revaluation of and engagement with Indigenous Peoples and knowledge systems. The findings from this study show that not all nursing faculty and programs are at the same place on the continuum. Nevertheless, the evidence from the data analyses also suggests that, although change is happening slowly, the nursing academy is oriented towards a long‐term goal of transformation; the extent of this transformation remains to be seen.

## Funding

The author received no specific funding for this work.

## Ethics Statement

The study received harmonized Research Ethics Board approval through the University of Regina (REB # 2021‐035), with reciprocal approvals through the University of Saskatchewan and Saskatchewan Polytechnic.

## Conflicts of Interest

The author declares no conflicts of interest.

## Data Availability

The data that support the findings of this study are available on request from the corresponding author. The data are not publicly available due to privacy or ethical restrictions.

## Footnotes

1 The term *Sixties Scoop* was coined by Patrick Johnston, author of the 1983 report *Native Children and the Child Welfare System* (McKenzie et al. 2016). The Sixties Scoop was the name given to the policy of mass apprehension of Indigenous children from their families and into the care of child welfare, including placing them in non‐Indigenous homes. This occurred over a period of over 20 years and formally ended in the mid‐1980s (Allan and Smylie 2015; Fournier and Crey 1997).

2 Epistemic violence is a form of injustice that occurs when one is unfairly denied the ability to be recognized as a credible knower, often due to prejudice based on cultural or social identity (Fricker 2007).

3 National Council Licensure Examination (NCLEX) is an entry‐to‐practice examination that tests the competencies that nurses should demonstrate at the beginning of their careers.
